# Reconstruction of full thickness wounds using glyaderm in a single-staged procedure

**DOI:** 10.1007/s10561-021-09907-x

**Published:** 2021-02-23

**Authors:** Melissa de Henau, Anne Sophie Kruit, Dietmar J. O. Ulrich

**Affiliations:** Department of Plastic and Reconstructive Surgery, Radboud University Medical Center, Geert Grooteplein Zuid 10, 6523 GA Nijmegen, The Netherlands

**Keywords:** Full thickness wound, Dermal substitute, Glyaderm, Skin graft, Negative pressure wound therapy

## Abstract

**Introduction:**

In large full-thickness skin defects, donor site morbidity limits the available thickness and surface of skin autografts and therefore only split-thickness skin grafts are possible for reconstruction. Dermal equivalents can be added to these split-thickness grafts to acquire an anatomically better skin reconstruction. Glyaderm is a human derived, acellular dermis and up until now has only been used in a two-staged procedure. This report describes results of a case series using Glyaderm and split-thickness skin grafts in a single-staged procedure.

**Methods:**

Glyaderm was introduced in 2017 in Radboudumc (Nijmegen, The Netherlands). Glyaderm and autologous split-skin grafts were simultaneously applied to the wounds. In cases with large wound surfaces or wounds covering highly mobile areas, negative pressure wound therapy was additionally applied. The first ten cases were followed with regular intervals post-operatively, assessing graft take, scar appearance, post-operative wound problems and re-interventions.

**Results:**

Patients were aged 3 weeks to 76 years-old. Treated skin surface varied from 1–16% total body surface. Wounds resulted from trauma (n = 4), burns (n = 4) or soft tissue infections (n = 2). Follow-up varied from 4 months to 1.5 years. No complications occurred after surgery. Average take rate was 98%. Two patients had a later re-intervention to further improve the aesthetic appearance of the scarred area.

**Conclusion:**

Our first results with the application of Glyaderm in a single-staged procedure provided good healing, graft take and scar appearance. Glyaderm was found a suitable dermal substitute in the treatment of full thickness wounds.

## Introduction

One of the main treatment options for skin defects is split-thickness skin grafting (STSG). However, when the surface and depth of the skin defect is extensive, such as in severely burned patients, donor site morbidity limits the available thickness and surface of skin autografts. This necessitates STSG with meshing and expansion of the grafts, resulting in unaesthetic healing and may lead to hypertrophic scars.

In the past two decades, the concept of a bi-layered wound coverage in the treatment of extensive full thickness wounds has become widely accepted (Demling et al. 2007; Nguyen et al. 2010). In this concept, a dermal substitute is used in combination with a STSG. The combined use of a dermal and epidermal analog mimics normal skin anatomy and may therefore improve aesthetic outcome and diminish scar hypertrophy (Pirayesh et al. 2007).

A dermal substitute can be defined as a bio-matrix that fulfills the functions of the normal cutaneous dermal layer. The general requirements of a dermal substitute are protecting the wound from infection and fluid loss and providing a stable and biodegradable template that improves the synthesis of new dermal tissue. The dermal substitute thus provides a scaffold during the healing process to the cells infiltrating the wound bed to promote tissue growth and to enhance wound healing (Lee 2000). This results in newly formed dermal tissue rather than scar tissue which is better able to resist tear forces and is more elastic and thus less painful (van der Veen et al. 2010).

Several dermal substitutes have become available, derived from synthetic or biological materials. The biological materials can either be derived from allogeneic material (human) or xenogeneic material (mainly porcine and bovine). Biological dermal substitutes show high similarity to native dermis and provide a 3-dimensional extra-cellular matrix of collagen and elastin without cells (Shahrokhi et al. 2014). To date, the biological derivates are preferred in clinical practice (Truong et al. 2005; Shahrokhi et al. 2014).

The use of the first human-derived dermal substitute, AlloDerm® was described in 1995 (Wainwright 1995). AlloDerm® is made of human cadaver skin that has been chemically treated in multiple stages to remove all donor cells. Good results have been described on its use, however, the production of AlloDerm® is a complex process and comes at high costs (Reported price in 2013; €21.7/cm^2^; Butterfield 2013).

In 2008, Richters et al. developed a cost-efficient technique to create an acellular dermal matrix from glycerol preserved allogeneic skin (Richters et al. 2008). The resulting non-profit dermal substitute is available as Glyaderm (Glycerol preserved Acellular Dermis) by the Euro Tissue Bank (ETB-BISLIFE, Skin Department, Beverwijk, The Netherlands). The use of glycerol preservation and Na-OH incubation removes all antigenetic structures and cells.

To date, treatment of full thickness defects with Glyaderm has only been described as a two-staged procedure. In the first stage, Glyaderm is applied to a granulating wound bed and covered by sterile dressings. The second stage, split thickness autografting, is usually performed 5–7 days later (Pirayesh et al. 2015). This protocol has shown to have good clinical outcomes, with better scar quality and aesthetic outcomes compared to STSG alone. The main disadvantage of this protocol is the use of two stages. Glyaderm was introduced in 2017 in the Radboudumc (Nijmegen, the Netherlands) to aid closure of selected full thickness wounds, for instance wounds overlying a joint surface or with a large surface. A new protocol was used, were Glyaderm was applied in a single-staged procedure. This article describes the results of the first ten cases.

## Materials and methods

Ten patients who presented in the Radboud University Medical Center between 2017 and 2019 with full thickness defects of various origins were treated with Glyaderm and STSG in a single-stage procedure (Table 1). When a healthy wound bed was reached after debriding, the wounds were covered in one session with Glyaderm (average thickness 0.3 mm) and STSG from various donor sites, depending on the localization of the wound. Following the application and fixation of Glyaderm to cover the entire wound, the STSG was applied on top of it. Both layers were fixated using absorbable sutures. In seven cases with large wound surfaces or in wounds spanning highly mobile areas, negative pressure wound therapy (NPWT) was applied immediately after wound coverage intra-operatively. The NPWT was used continuously at a pressure of − 125 mmHg until the first wound inspection at 5 days post-operative. After removal of the NPWT device, wounds were covered with paraffin gauzes until a stable skin ingrowth was reached. In the three remaining cases where no NPWT was applied, wounds were covered with paraffin gauzes and a tie-over until the first wound inspection. Graft take rate was evaluated at 5–7 days, 2 months and every 6 months thereafter by clinical evaluation. Scar aesthetic appearance and hypertrophy were evaluated and recorded by the threating physician.

**Table 1:** Patient characteristics and outcomes

Pt no	Age (years)	Gender	Date of surgery	Defect area	Defect cause	TBSA (%)	Take rate (%)	NPWT (days)
1	19	Female	2017-03-03	Left flank	Infection	16	96	5
2	28	Female	2017-08-02	Amputation stump left upper leg	Trauma	6	98	5
3	77	Female	2017-08-07	Right lower leg	Grade 3 burn	3	100	–
4	22	Male	2017-08-08	Left dorsal wrist	Trauma	1	99	–
5	25	Male	2018-02-12	Lower back	Grade 2–3 burn	11	99	5
6	26	Male	2018-06-12	Thorax	Grade 2–3 burn	6	99	–
7	76	Male	2018-10-31	Right leg	Trauma	16	96	5
8	6	Male	2018-10-20	Left knee region	Deep grade 2 burn	2	100	5
9	3 week	Female	2019-05-27	Lower back	Trauma	7	90	5
10	3 week	Female	2019-06-24	Buttocks and dorsal upper legs	Infection	10	98	5

*TBSA* total body surface area, *NPWT* negative pressure wound therapy

## Case presentation

### Case 1

**Fig. 1:**
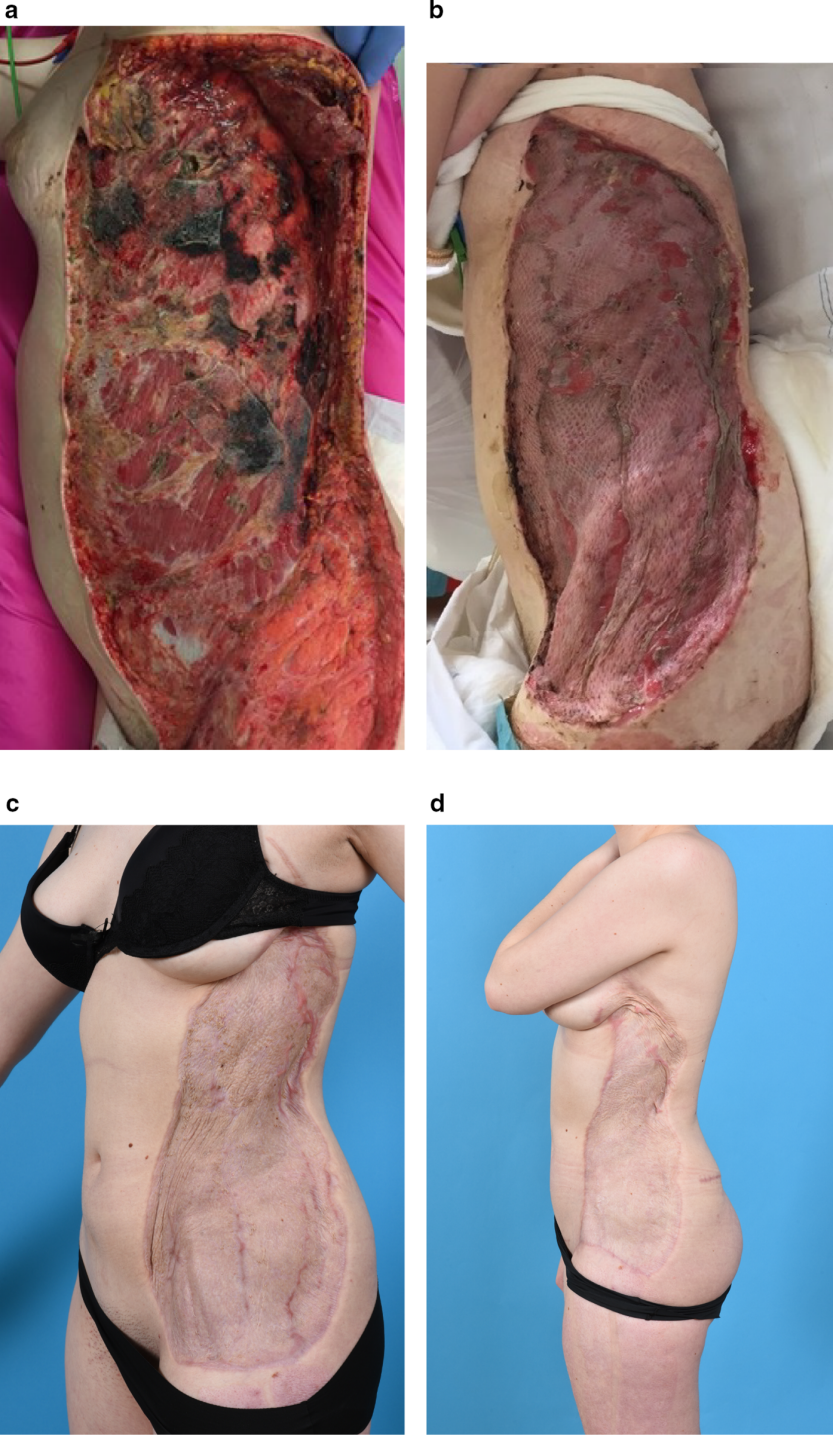
Case 1. **a** Before wound coverage (preparation of the wound bed with NPWT); **b** 10 days after Glyaderm + STSG application; **c** 6 months after Glyaderm + STSG application; **d** 1.5 year after Glyaderm + STSG application (after skin expansion and partial scar resection medially)

Our first case was a 19-year-old healthy girl who suffered necrotizing fasciitis in February 2017. This resulted from a complicated tonsillitis with left-sided pleural empyema and thorax drainage. The necrotizing fasciitis developed around the drain insertion. The skin and fascia of her left flank were excised in multiple sessions to reach a healthy wound bed. This resulted in a wound with an affected total body surface area (TBSA) of 12% (900cm^2^) (Fig. 1). One week after the initial debridement, the wound was covered in one session with Glyaderm and STSG taken from both upper legs. A negative pressure wound therapy (NPWT) device was applied over the grafts to aid in ingrowth and was left in place for 5 days. The graft take rate was 96%. After 6 months, she developed an elastic skin and the mesh pattern faded nicely. She had a slight tight feeling at her wound and there was a small hypertrophic area at the cranial side of the wound. At 1.5 year, the aesthetic appearance of the wound further increased with natural color and smoother transition of scar to native skin at the wound edges. Nevertheless, her scar is currently being reconstructed in multiple sessions by using tissue expansion, aiding in coverage of the scarred area with native skin.

### Case 2

**Fig. 2:**
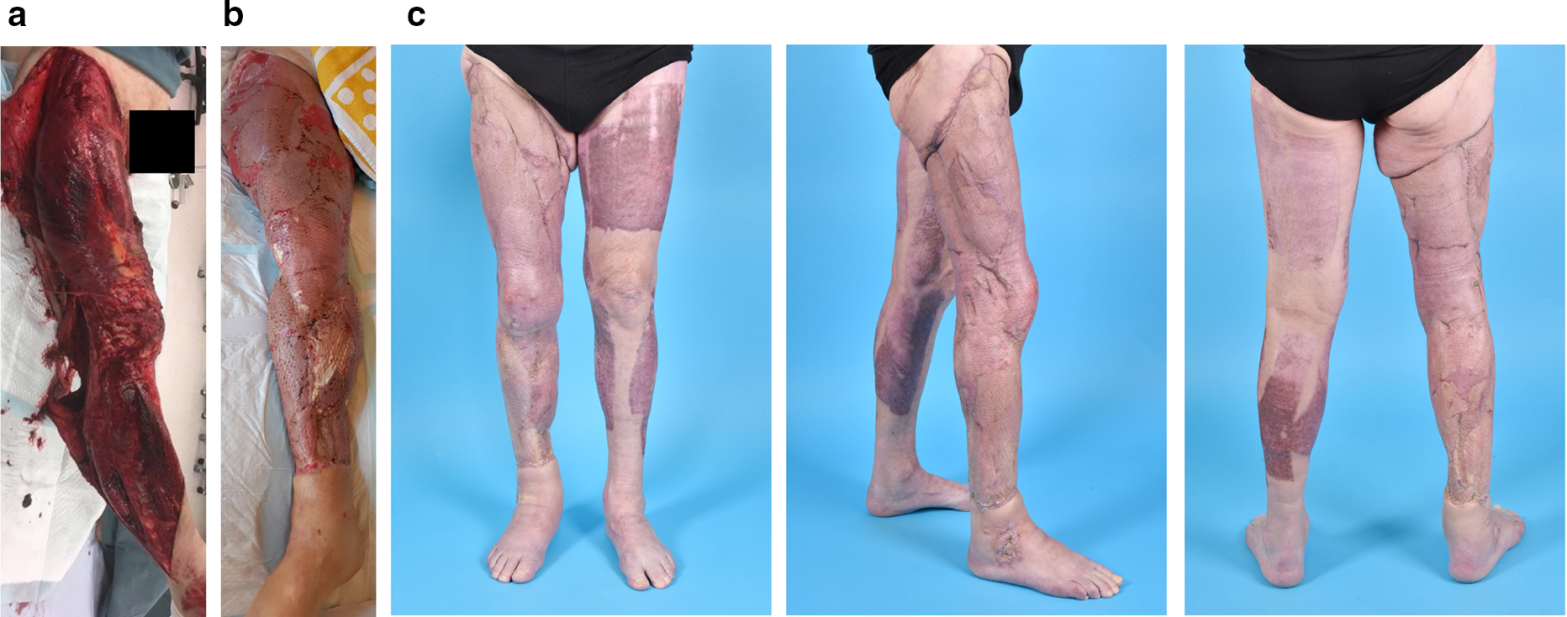
Case 2. **a** Immediately after trauma; **b** 2 weeks after Glyaderm + STSG application; **C** 5 months after Glyaderm + STSG application

A 76-year-old healthy male was brought in with a severe deglovement injury of his entire right leg in a low-speed accident with a truck (TBSA 16%) (Fig. 2). He had multiple fractures of his fibular head, patella and distal tibia, without neurovascular damage. He was immediately taken for surgery, with removal of necrotic skin and fasciotomy. At a second and third look, additional necrotectomies were performed and a NTWP device was placed. Three weeks later there was a stable situation and the defect was ready for coverage. A local hemisoleus muscle flap was used to cover a deep defect at the proximal tibial area, followed by Glyaderm + STSG coverage of the entire leg in a single procedure. The STSG donor sites were the left leg and abdomen. Average take after 2 weeks was 98.5%. At 5 months postoperatively, the wound was fully closed and the mesh pattern started to fade. There was a tightness around the knee that restricted knee flexion to 60 degrees, for which an intensive physical therapy program was followed until a maximum of 95 degrees’ flexion was reached. Further follow-up is planned to decide whether additional reconstruction is required to improve knee mobility.

## Outcomes and follow-up

Ten patients were treated with Glyaderm and STSG in a single-stage procedure between 2017 and 2019 (Table 1). Patients were aged 3 weeks to 76 years old. Indications were: burns (n = 4), traumatic wounds (n = 4) and skin defects after soft tissue infections (n = 2). The treated skin surface varied from 1 to 16% TBSA. The smaller defects were on hands or overlying joints, were skin elasticity is of utmost importance for function. In seven cases with large wound surfaces or in wounds spanning highly mobile areas, NPWT was applied immediately after wound coverage. Follow-up varied from 4 months to 1.5 years. No complications occurred after surgery. The mean take rate was 98%. Two cases showed mild hypertrophy at the scar edges and two patients required a later re-intervention to further improve the scar aesthetic appearance, amongst which our first case.

## Discussion

In 2008, Glyaderm was developed as a cost-efficient alternative for human acellular dermal substitutes (Richters et al. 2008). The price for Glyaderm being €4.60/cm^2^ in 2020. The de-cellularization method with NaOH on glycerol preserves the donor elastin fibers well. These fibers are important for the ingrowth of host fibroblast and blood vessels and function as a “guide” for the fibroblasts in the turnover of donor collagen into host collagen, resulting in a more natural newly formed dermis with assumingly better elasticity (Pirayesh et al. 2015).

In a pilot study Pirayesh et al. (2015) a two-staged reconstruction with Glyaderm + STSG was compared to STSG alone. The mean Glyaderm + STSG take rate was 91.55% and mean STSG alone take rate was 96.67%. Although there was no statistically significant difference in take rate, the authors found a better elasticity of scars in the Glyaderm group. Objective measurements of scar color and pigmentation were not statistically different between the two groups, as were the subjective scar scales (Pirayesh et al. 2015).

To our knowledge, Glyaderm has only been described in a two-staged procedure. In the first stage, Glyaderm is applied to a prepared wound bed, followed by the STSG autograft application 5–7 days later. Immediate application of Glyaderm to an unprepared wound bed was initially attempted, but showed a lack of ingrowth of Glyaderm, even after meshing it (Pirayesh et al. 2015). Since then, improvements have been made to the technical processing and adequate graft selection to obtain dermis’ of uniform thickness, resulting in thinner and qualitatively more consistent dermal matrices. A thinner acellular dermal matrix improves vascular ingrowth possibilities from the granular wound bed to the STSG, providing it with oxygen and nutrients and increasing graft take. This article presents a case series were Glyaderm and STSG autografts were successfully applied in a single-staged procedure.

In this case series, some wounds were covered with a NPWT device after Glyaderm + STSG application. NPWT has been used since centuries to treat wounds (Miller 2012). NPWT in its most current form is used to heal complex wounds by draining excessive wound exudate and increasing the vascularity of the wound bed, resulting in increased granulation tissue formation compared to standard wound dressing (Argenta and Morykwas 1997; Sinha et al. 2013). Combining NPWT and dermal substitutes has already been studied in the past with good results (Molnar et al. 2004; Hutchison and Craw 2013). Liu et al. compared the use of acellular dermal matrix (ADM) with or without NPWT in treatment of deep burn wound in porcine limbs and found superior results in the ADM + NPWT group (Liu et al. 2016). Advantages of combining NPWT and dermal substitutes are less seroma formation, prevention of excessive movement during STSG ingrowth, easy wound care, good patient tolerance and consistent vascularization.

This is the first report describing the use of Glyaderm and STSG as a single-staged procedure, combined with or without NPWT treatment. Our cases show good results and suggest that bi-layered wound reconstruction with Glyaderm + STSG is feasible in a single-stage procedure. Our sample size is too small to draw definite conclusions and randomized controlled trials with larger patient numbers are needed to further assess results compared to STSG alone.

## Take home messages

- An acellular dermal matrix serves as a collagen/elastin scaffold to guide ingrowth of new dermal tissue
- Double-layered reconstruction might be recommended in areas overlying joints or in large wounds
- Glyaderm is of human origin and has lower costs compared to other biological ADMs
- A single-staged procedure for applying Glyaderm + STSG with or without NPWT is feasible and shows good clinical results in our first cases
- NPWT can be safely used in this procedure
